# RNA sequencing reveals induction of specific renal inflammatory pathways in a rat model of malignant hypertension

**DOI:** 10.1007/s00109-021-02133-8

**Published:** 2021-09-15

**Authors:** Carlos Menendez-Castro, Nada Cordasic, Fabian B. Fahlbusch, Arif B. Ekici, Philipp Kirchner, Christoph Daniel, Kerstin Amann, Roland Velkeen, Joachim Wölfle, Mario Schiffer, Andrea Hartner, Karl F. Hilgers

**Affiliations:** 1 Department of Pediatrics and Adolescent Medicine, University Hospital Erlangen, Friedrich-Alexander University of Erlangen-Nürnberg, Erlangen, Germany; 2 Department of Nephrology and Hypertension, University Hospital Erlangen, Friedrich-Alexander University of Erlangen-Nürnberg, Ulmenweg 18, 91054 Erlangen, Germany; 3 Institute of Human Genetics, University Hospital Erlangen, Friedrich-Alexander University of Erlangen-Nürnberg, Erlangen, Germany; 4 Institute of Nephropathology, University Hospital Erlangen, Friedrich-Alexander University of Erlangen-Nürnberg, Erlangen, Germany

**Keywords:** Malignant hypertension, Two-kidney one-clip renovascular hypertension (2K1C), Kidney injury, Inflammation, Complement activation, RNA-Seq

## Abstract

**Abstract:**

In malignant hypertension, far more severe kidney injury occurs than in the “benign” form of the disease. The role of high blood pressure and the renin–angiotensin–aldosterone system is well recognized, but the pathogenesis of the renal injury of malignant hypertension (MH) remains incompletely understood. Using the rat model of two-kidney, one-clip renovascular hypertension in which some but not all animals develop MH, we performed a transcriptomic analysis of gene expression by RNA sequencing to identify transcriptional changes in the kidney cortex specific for MH. Differential gene expression was assessed in three groups: MH, non-malignant hypertension (NMH), and normotensive, sham-operated controls. To distinguish MH from NMH, we considered two factors: weight loss and typical renovascular lesions. Mean blood pressure measured intraarterially was elevated in MH (220 ± 6.5 mmHg) as well as in NMH (192 ± 6.4 mmHg), compared to controls (119 ± 1.7 mmHg, *p* < 0.05). Eight hundred eighty-six genes were exclusively regulated in MH only. Principal component analysis revealed a separated clustering of the three groups. The data pointed to an upregulation of many inflammatory mechanisms in MH including pathways which previously attracted relatively little attention in the setting of hypertensive kidney injury: Transcripts from all three complement activation pathways were upregulated in MH compared to NMH but not in NMH compared with controls; immunohistochemistry confirmed complement deposition in MH exclusively. The expression of chemokines attracting neutrophil granulocytes (CXCL6) and infiltration of myeloperoxidase-positive cells were increased only in MH rats. The data suggest that these pathways, especially complement deposition, may contribute to kidney injury under MH.

**Key messages:**

- The most severe hypertension-induced kidney injury occurs in malignant hypertension.
- In a rat model of malignant hypertension, we assessed transcriptional responses in the kidney exposed to high blood pressure. A broad stimulation of inflammatory mechanisms was observed, but a few specific pathways were activated only in the malignant form of the disease, notably activation of the complement cascades.
- Complement inhibitors may alleviate the thrombotic microangiopathy of malignant hypertension even in the absence of primary complement abnormalities.

**Supplementary Information:**

The online version contains supplementary material available at 10.1007/s00109-021-02133-8.

## Introduction

Arterial hypertension is one of the major risk factors for the development of cardiovascular disease [1]. Additionally, chronic elevation of blood pressure can induce chronic kidney disease (CKD) [2]. Vice versa, renal disease causes arterial hypertension [3]. Malignant hypertension is characterized by a marked elevation of blood pressure and the occurrence of progressive end-organ damage (e.g., renal, cerebral, vascular, or ocular injury) [4, 5]. In the kidney, malignant hypertension leads to a form of thrombotic microangiopathy, exhibiting fibrinoid necrosis and proliferative lesions of the small arteries [6] accompanied by kidney fibrosis and loss of renal function [7].

The pathomechanistic factors for the development of malignant hypertension in contrast to the development of non-malignant arterial hypertension are only incompletely understood. In animal models, a marked and rapid rise of blood pressure appears to be a necessary condition [8]. On the other hand, blood pressure differences between malignant hypertensive animals and appropriate non-malignant hypertensive controls can be quite small [9, 10]. Griffin et al. reported that even a small reduction of blood pressure alleviated renal injury in a rat model of malignant hypertension, leading to the concept of a critical pressure threshold for malignant hypertension [5]. Several studies indicated that the activation of the renin–angiotensin–aldosterone system (RAAS) might play an important role [9, 11, 12]. A chronic activation of RAAS was also associated with the induction of inflammatory pathways [9, 13]. Furthermore, in a rat model of renovascular hypertension, malignant hypertension was accompanied by an impaired neovascularization and reduced capillary supply in heart and kidney tissue [14]. This might contribute to the specific vascular lesions and progressive organ failure seen in the kidney under malignant hypertension.

Here, we used a transcriptomic approach to elucidate specific alterations of gene expression of the kidney cortex in malignant versus non-malignant hypertension. We performed high-throughput RNA sequencing (RNA-seq) in a rat model of two-kidney, one-clip renovascular hypertension (2K1C) [15]. Thirty-five days after clipping, differential gene expression in renal cortex was assessed in three animal groups: rats with malignant hypertension (MH), rats with non-malignant hypertension (NMH), and normotensive, sham-operated controls (sham). We focused on the non-clipped kidney exposed to hypertension, because it is the non-clipped kidney, which exhibits the characteristic vascular lesions of malignant nephrosclerosis. Several identified candidate genes were further assessed by RT-PCR and immunohistochemistry.

## Results

### Malignant hypertension

As described previously, the rat model of 2K1C was used to induce renovascular hypertension. Over a period of 35 days, only a part of rats spontaneously developed malignant hypertension (MH) after clipping of the left renal artery with a silver clip of 0.2 mm internal diameter [15]. MH animals were classified by significantly reduced postinterventional weight gain and the occurrence of fibrinoid necrosis and onion skin type lesions as characteristic vascular lesions in the contralateral kidney (supplementary Fig. 1) as described earlier [14]. Non-malignant hypertensive (NMH) individuals in contrast were hypertensive without developing characteristic renal vascular lesions and showed normal weight gain (supplementary table 1). In MH animals increased serum levels of creatinine, urea and aldosterone were found compared to NMH and sham (supplementary table 1). Serum creatinine in MH increased to levels comparable to those observed in models of glomerulonephritis or subtotal nephrectomy [16, 17], as did albuminuria (supplementary table 2). Mean arterial blood pressure (MAP) values were higher in MH compared to NMH, while MAP of both NMH and MH was significantly increased compared to sham animals (supplementary table 1). Systolic blood pressure values obtained by sequential tail cuff measurements from week 1 until week 5 after 2K1C revealed a trend towards higher pressure levels in MH beginning 1.5 weeks post intervention (supplementary Fig. 2). Relative left ventricular weight was increased in MH and NMH animals compared to sham but did not significantly differ between MH and NMH (supplementary table 1) although there were vascular lesions and microinfarctions in the myocardium of MH (supplementary Fig. 3).

### Renal gene expression patterns in malignant hypertension

**Fig. 1:**
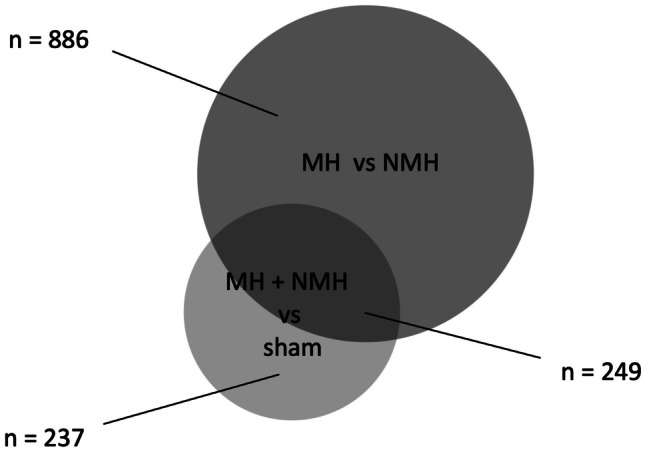
Venn diagram showing the number of differentially expressed genes from RNA-seq analysis

**Fig. 2:**
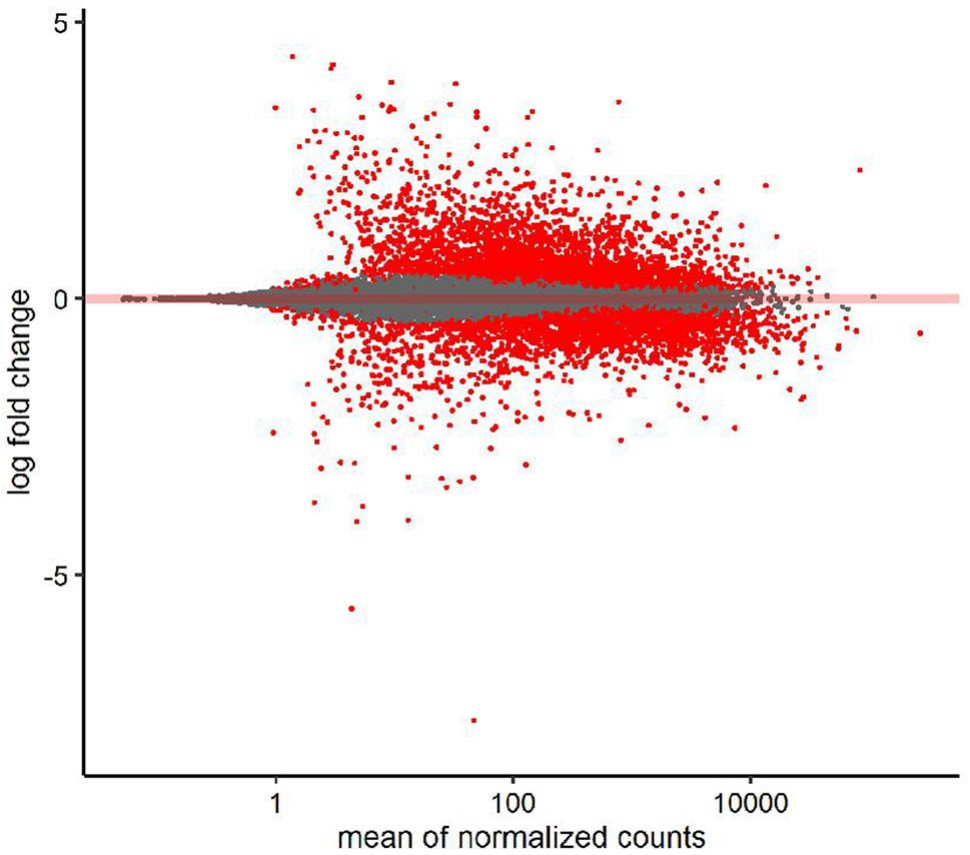
MA plot. Scatterplot of log2 expression fold changes versus mean gene expression. Fold changes from highly variable genes are compressed using the apeglm approach from DESeq2 to aid with interpretation. Genes with significant expression differences (adjusted *p*-value < 0.01) are shown in red

Global gene expression in the non-clipped right kidney was assessed in sham, MH, and NMH animals using mRNA-seq analysis [18]. To evaluate specific effects of malignant hypertension, differentially expressed genes in renal tissue were analyzed comparing MH and NMH animals. One thousand one hundred thirty-five genes showed a significantly differential expression (fold change ≤ 2, fold change ≥ 2, adjusted p-value < 0.01). Among them, 886 genes exclusively showed an expressional regulation in MH animals only (Fig. 1). Graphical representation of log2 fold change versus mean expression in MH and NMH animals indicated that the majority of regulated genes were upregulated (Fig. 2). Further evaluation of RNA-seq data comparing MH and NMH animals showed 765 genes significantly upregulated and 370 genes significantly downregulated in MH.

**Fig. 3:**
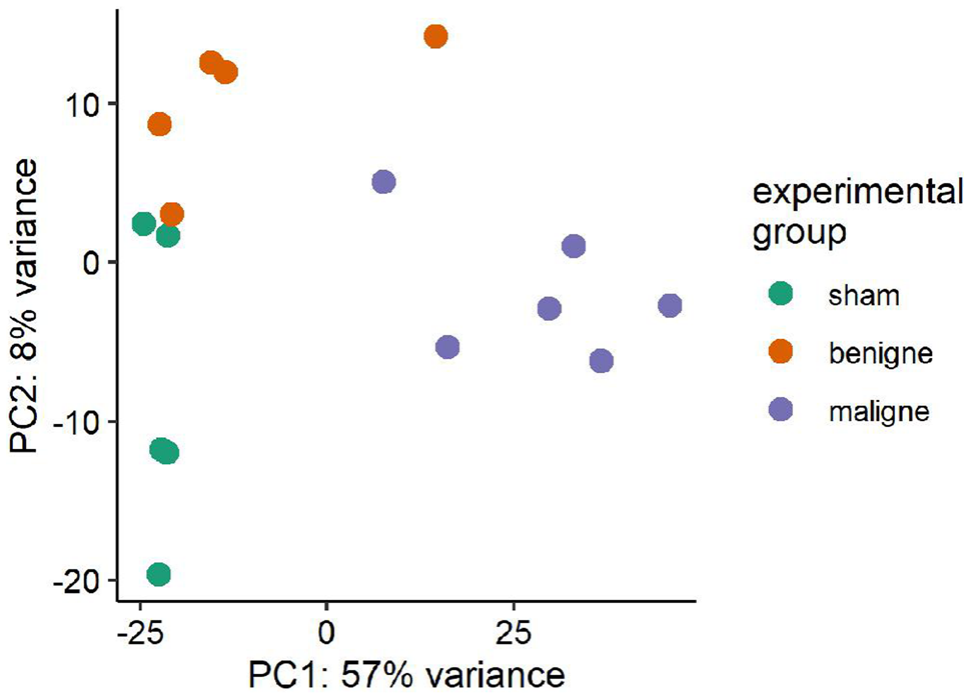
Principal component analysis (PCA) of gene expression in the individual samples. The labeling of the axes indicates the percentage of total variance explained by each component. Due to the higher variance explained by the PC1, differences along the x-axis are larger compared to differences along the y-axis. PC, principal component

**Fig. 4:**
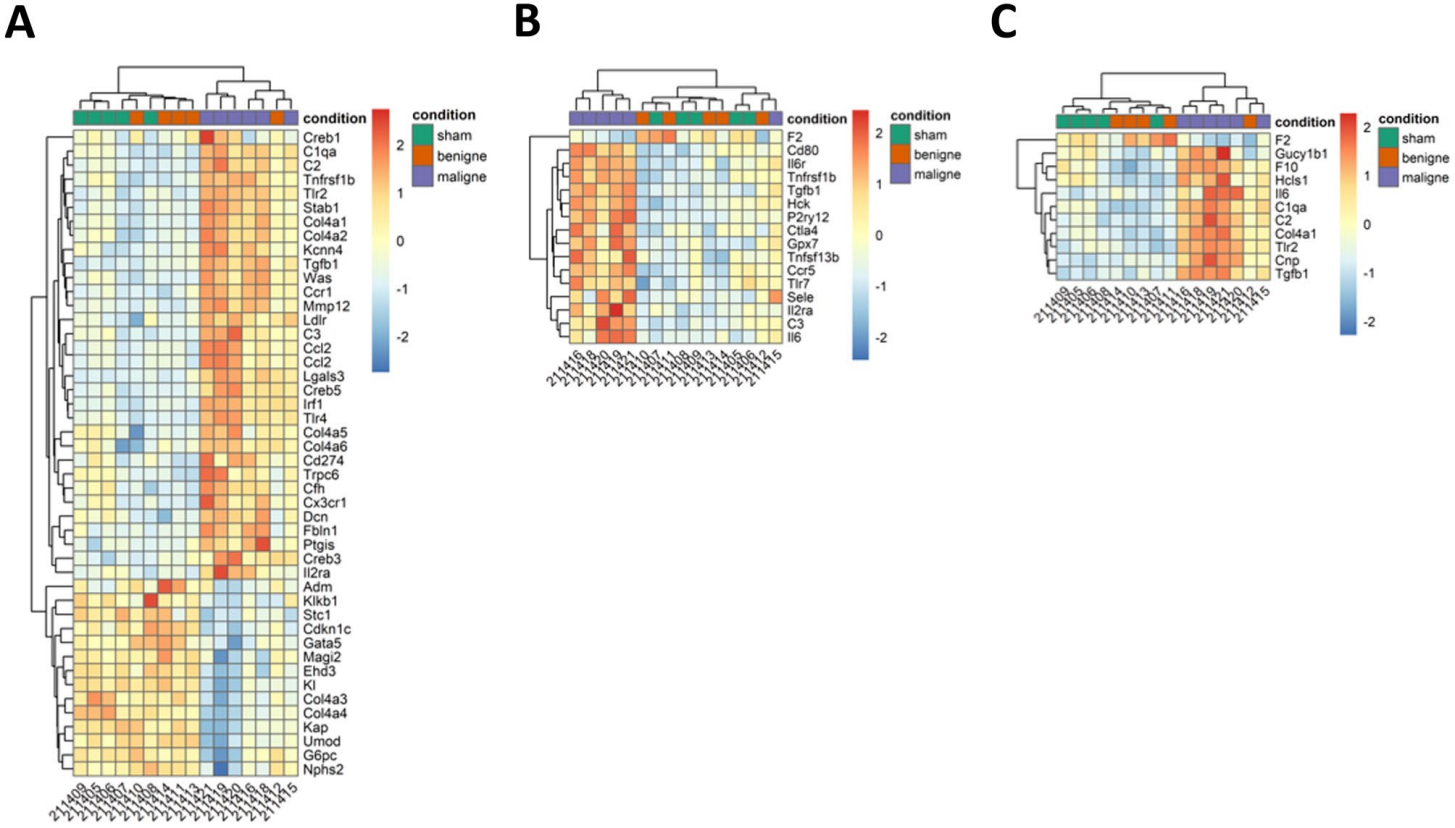
Heat map analyses. Heat maps of normalized (rlog, DESeq2) expression values, centered and scaled by row. Clustering uses the Euclidean distance with complete linkage. **A** Heat map with genes related to “glomerular disease”; **B** heat map with genes related to “vasculitis”; **C** heat map with genes related to “thrombosis”

Principal component analysis (PCA) revealed a separated clustering of the three study groups (Fig. 3). This observation is also supported by selected heat maps, depicting the differential expression of target genes related to “glomerular disease” (Fig. 4A), “vasculitis” (Fig. 4B), and “thrombosis” (Fig. 4C) (supplementary Fig. 4 shows the same data ordered by predetermined experimental group). These three topic areas were chosen because they reflect major sequelae of malignant hypertension in the kidney—elevated blood pressure induced glomerular and vascular changes and thrombotic microangiopathy.

### Inflammatory pathways

Ingenuity pathway analysis (IPA, Qiagen) was used for further in silico network and pathway analysis of differentially expressed genes. Disease-specific comparison of MH and NMH animals revealed “inflammatory response” as the most significantly altered signaling network based on the associated number of differentially regulated genes (Table 1). Accordingly, IPA software identified the most relevant upstream regulators to belong to the group of proinflammatory molecular markers (Table 2).

**Table 1:** Top 5 diseases and disorders

	***p*-value range**	**Number of molecules**
Inflammatory response	6,03E-13—2,02E-51	412
Endocrine system disorders	7,15E-16—9,92E-47	215
Gastrointestinal disease	2,28E-13—9,92E-47	909
Metabolic disease	7,15E-16—9,92E-47	236
Organismal injury and abnormalities	8,19E-13—9,92E-47	973

**Table 2:** Top 5 upstream regulators

	***p*-value**
TNF	2,96E-57
TGFB1	8,88E-43
IFNG	1,25E-40
IL1B	2,07E-40
CSF2	5,48E-35

**Fig. 5:**
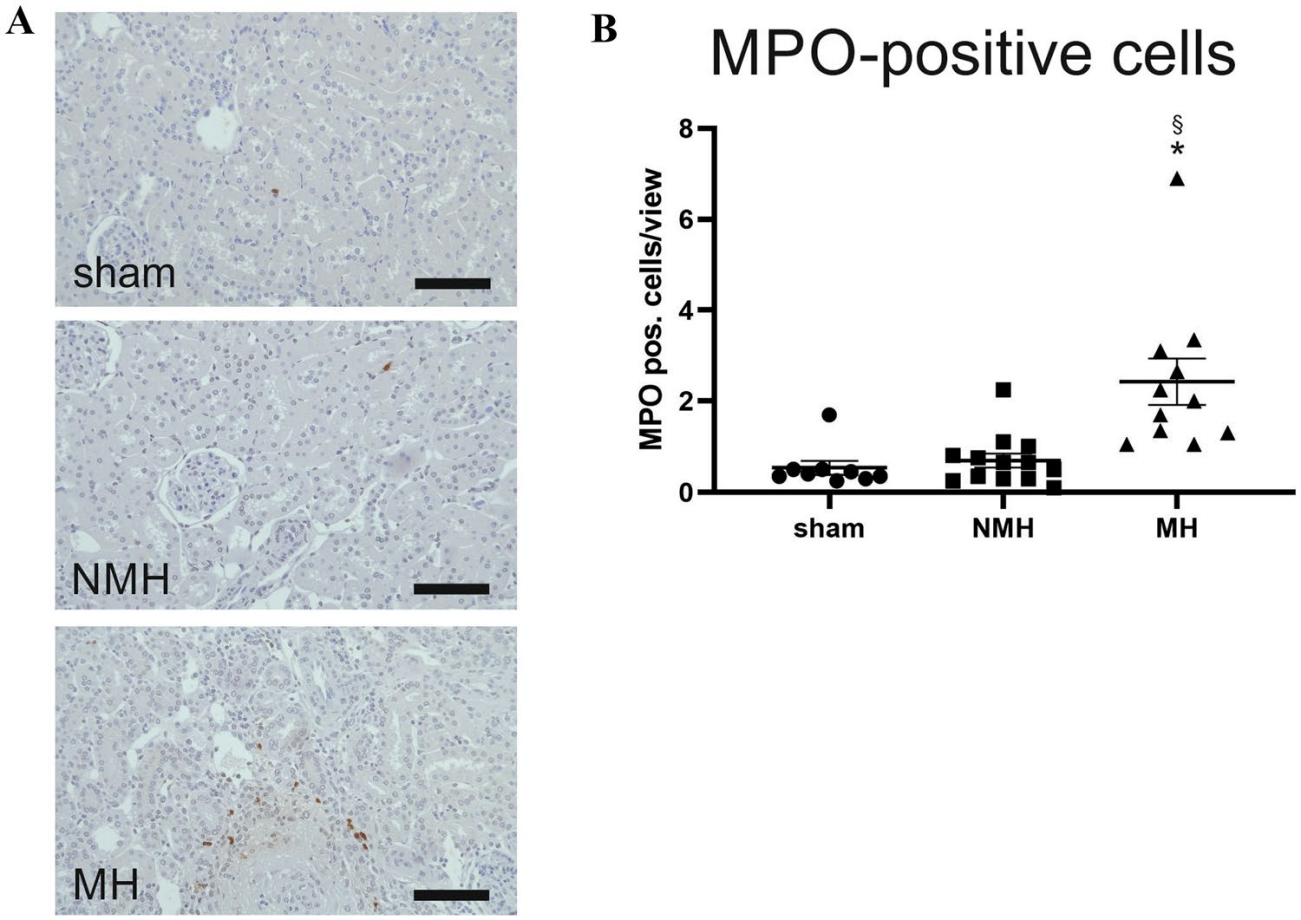
Infiltration of MPO-positive cells into kidney tissue. **A** Exemplary photomicrographs of renal sections stained for myeloperoxidase (MPO). Bar represents 100 μm. **B** Evaluation of MPO-positive cell counts. Sham, sham-operated animals (*n* = 9); NMH, non-malignant hypertension (*n* = 13); MH, malignant hypertension (*n* = 11). Data are means ± standard error of the mean, statistical test one-way ANOVA, Bonferroni post hoc test. * *p* < 0.05 vs. sham, § *p* < 0.05 vs. NMH

Based on these findings, a further assessment of inflammatory cells in the kidney was performed. Using immunohistochemistry, more cytotoxic T-cells were found in the kidneys of MH animals compared to controls (Table 3, supplementary Fig. 5), while numbers of T-helper cells were only increased in NMH animals compared to controls (Table 3, supplementary Fig. 5). The local quantity of renal M1 macrophages was significantly higher in MH animals compared to NMH and sham animals (Table 3, supplementary Fig. 5). However, the number of M2 macrophages was increased only in the kidneys of MH animals compared to sham-controls (Table 3, supplementary Fig. 5). A significantly higher infiltration by MPO-positive cells was found in MH compared to NMH and sham (Table 3, Fig. 5). Macrophages as well as neutrophil granulocytes may be MPO-positive; stainings of serial sections for MPO and CD68 showed that at least part of the MPO-positive cells were CD68 negative (supplementary Fig. 6), suggesting infiltration of neutrophil granulocytes in MH. Fox-P3-positive regulatory T cells were increased in MH and NMH versus sham-operated controls, but there was no difference between MH and NMH, respectively (supplementary Fig. 7).

**Table 3:** Inflammatory cells, chemokines, and cytokines

	**Sham**	**NMH**	**MH**
**Inflammatory cells**			
MPO-positive cells [cells/view]	0.53 ± 0.15	0.69 ± 0.15	2.43 ± 0.51*^§^
M1 macrophages [CD68-positive cells/view]	5.7 ± 0.53	8.87 ± 0.82*	14,66 ± 1.44*^§^
M2 macrophages [Cd163-positive cells/view]	0.09 ± 0.04	0.58 ± 0.24	1.19 ± 0.3*
T-helper cells [Cd4-positive cells/view]	11.56 ± 3.37	88.15 ± 22.7*	57.11 ± 13.45
Cytotoxic T-cells [Cd8a-positive cells/view]	3.12 ± 0.38	4.2 ± 0.45	5.46 ± 0.47*
**Chemokines**			
CCL2 [fold induction]	1.00 ± 0.23	5.85 ± 1.46	11.48 ± 1.33*^§^
CCL3 [fold induction]	1.00 ± 0.14	2.15 ± 0.5	2.70 ± 0.31*
CCL5 [fold induction]	1.00 ± 0.32	1.66 ± 0.23	2.24 ± 0.43
CCL7 [fold induction]	1.00 ± 0.23	4.50 ± 0.65*	7.78 ± 0.95*^§^
CXCL3 [fold induction]	1.00 ± 0,32	4,85 ± 1,1	12,99 ± 5,7
CXCL6 [fold induction]	1.00 ± 0.32	14.38 ± 5.33	56.74 ± 13.43*^§^
CXCL8 [fold induction]	1.00 ± 0.18	1.58 ± 0.36	2.52 ± 0.57
CCR2 [fold induction]	1.00 ± 0.14	6.54 ± 2.62	11.7 ± 1.9*
**Cytokines**			
IL-6 [fold induction]	1.00 ± 0.24	12.67 ± 2.66*	19.24 ± 2.3*
IL-10 [fold induction]	1.00 ± 0.17	2.62 ± 0.47	5.90 ± 0.71*^§^
IL-17a [fold induction]	1.00 ± 0.12	5.40 ± 1.94	26.85 ± 10.5*
LIF [fold induction]	1.00 ± 0.16	8.51 ± 1.79*	19.16 ± 2.26*^§^
TNF-α [fold induction]	1.00 ± 0.21	2.54 ± 0.42*	4.00 ± 0.35*^§^
**Costimulatory molecules**			
CD80 [fold induction]	1.00 ± 0.18	4.83 ± 1.25	10.9 ± 2.27*^§^
CTLA4 [fold induction]	1.00 ± 0.22	4.47 ± 1.29	13.34 ± 2.79
**Adhesion molecules**			
ICAM-1 [fold induction]	1.00 ± 0.17	5.57 ± 1.63	9.73 ± 0.56*
VCAM-1 [fold induction]	1.00 ± 0.24	2.84 ± 0.62	4.08 ± 0.31*

^*^p-value < 0.05 versus sham, § *p*-value < 0.05 versus NMH

Renal expression levels of the chemokines CC-chemokine ligand 2 (CCL2), CC-chemokine ligand 7 (CCL7), and C-X-C motif chemokine ligand 6 (CXCL6) were significantly increased in the kidney of MH compared to NMH and sham animals (Table 3). CC-Chemokine ligand 3 (CCL3) expression was only increased in MH rats compared to controls. CC-chemokine ligand 5 (CCL5) and C-X-C motif chemokine ligand 3 (CXCL3) showed no induction under malignant hypertension (Table 3). The renal expression of C–C chemokine receptor type 2 (CCR2), the receptor of CCL2 and CCL7, showed an increased expression in MH compared to sham but not to NMH (Table 3).

In the group of cytokines, interleukin-10 (Il-10), leukemia inhibitory factor (LIF), and tumor necrosis factor-alpha (TNF-α) showed a significant expressional induction in MH compared to NMH and controls (Table 3). Interleukin-6 (Il-6) and interleukin-17a (Il-17a) expressions were increased only in MH compared to sham. CD80, a costimulatory molecule, showed an induction of renal expression in the kidney of MH compared to NMH, while for its ligand CTLA4, only a trend towards higher expression levels in MH animals was observed (Table 3). Vascular cell adhesion protein 1 (VCAM-1) and intercellular adhesion molecule 1 (ICAM-1) showed higher renal expression levels in MH compared to sham (Table 3).

Pearson’s correlation coefficient was used to quantify the association between selected molecular markers of inflammation and inflammatory cells (Table 4). CCL2 positively correlated with M1 and M2 macrophages. CCL7 only showed a positive correlation with M1 macrophages not with M2 macrophages. CXCL6 revealed a positive correlation with MPO-positive cells (Table 4).

**Table 4:** Correlation of selected molecular markers of inflammation and inflammatory cells

	***r***	***p*-value**
**CCL2** (mRNA expression) vs. **M1 macrophages** (cells/view)	0.82	< 0.001
**CCL2** (mRNA expression) vs. **M2 macrophages** (cells/view)	0.80	0.01
**CCL7** (mRNA expression) vs. **M1 macrophages** (cells/view)	0.83	< 0.001
**CCL7** (mRNA expression) vs. **M2 macrophages** (cells/view)	0.43	0.25
**CXCL6** (mRNA expression) vs. **MPO-positive cells** (cells/view)	0.83	< 0.001

*r* = Pearson’s correlation coefficient r

Statistical significance was defined as *p*-value < 0.05

A broad consistence was detected between RT-PCR and RNA-seq when comparing the expression changes of these candidate genes, except for CCL5 (Table 5).

**Table 5:** Overview of ratios for the relative expression changes in both analytic procedures (PCR and RNA-seq)

	**Ratio**	
**Gene**	**PCR (Mal vs. NMH)**	**RNA-seq (Mal vs. NMH)**
C3	2.12	3.62
C3aR1	1.56	3.72
C4b	3.52	6.86
C5aR1	2.95	3.09
C6	3.61	4.37
CCL2	1.96	3.45
CCL3	1.26	1.49
CCL5	1.35	0.65
CCL7	1.73	2.78
CXCL3	2.68	9.86
IL-6	1.52	3.5
IL-10	2.25	7.31
IL-17a	4.97	5.38
LIF	2.25	2.96
CD80	2.26	2.61
CTLA4	2.98	3.36
ICAM-1	1.75	2.20
VCAM-1	4.08	1.77

### Complement system

**Fig. 6:**
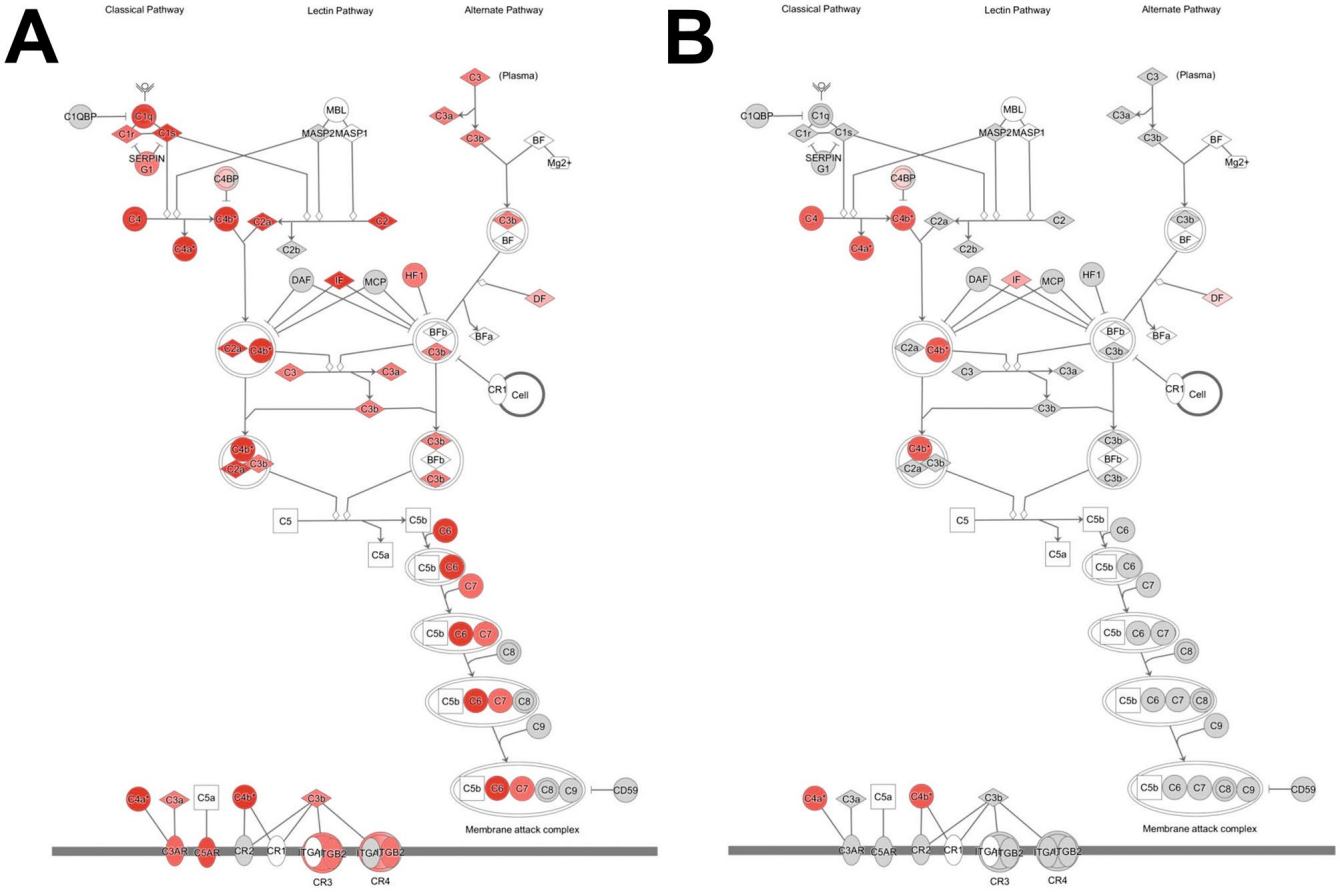
Schematic drawing of the complement cascade derived from ingenuity pathway analysis. All upregulated genes are depicted in red color. **A** MH vs. sham, **B** NMH vs. sham

RNA-seq analysis revealed a significant expressional induction of the classical complement system signaling pathway in MH compared to NMH animals (Fig. 6). Certain components of the lectin and alternate complement cascades were also activated. Using RT-PCR, the expressional induction of C4b, C5aR1, and C6 was verified in the kidneys of MH in comparison to NMH and sham. C3 and C3aR1 expression was found elevated in MH only compared to sham (Table 6). The expression of C3 was correlated with other complement components as well as with markers of infiltration and kidney injury but not with the level of blood pressure (supplementary table 3).

**Table 6:** mRNA expression of selected complement factors

	**Sham**	**NMH**	**MH**
C3 [fold induction]	1.00 ± 0.24	5.58 ± 1.71	11.84 ± 2.75*
C3aR1 [fold induction]	1.00 ± 0.22	2.87 ± 0.58	4.48 ± 0.90*
C4b [fold induction]	1.00 ± 0.14	8.85 ± 3.63	31.17 ± 7.10*^§^
C5aR1 [fold induction]	1.00 ± 0.21	3.46 ± 0.85	10.21 ± 2.25*^§^
C6 [fold induction]	1.00 ± 0.34	4.63 ± 1.46	16,72 ± 5.10*^§^

^*^p-value < 0.05 versus sham, § *p*-value < 0.05 versus NMH

**Fig. 7:**
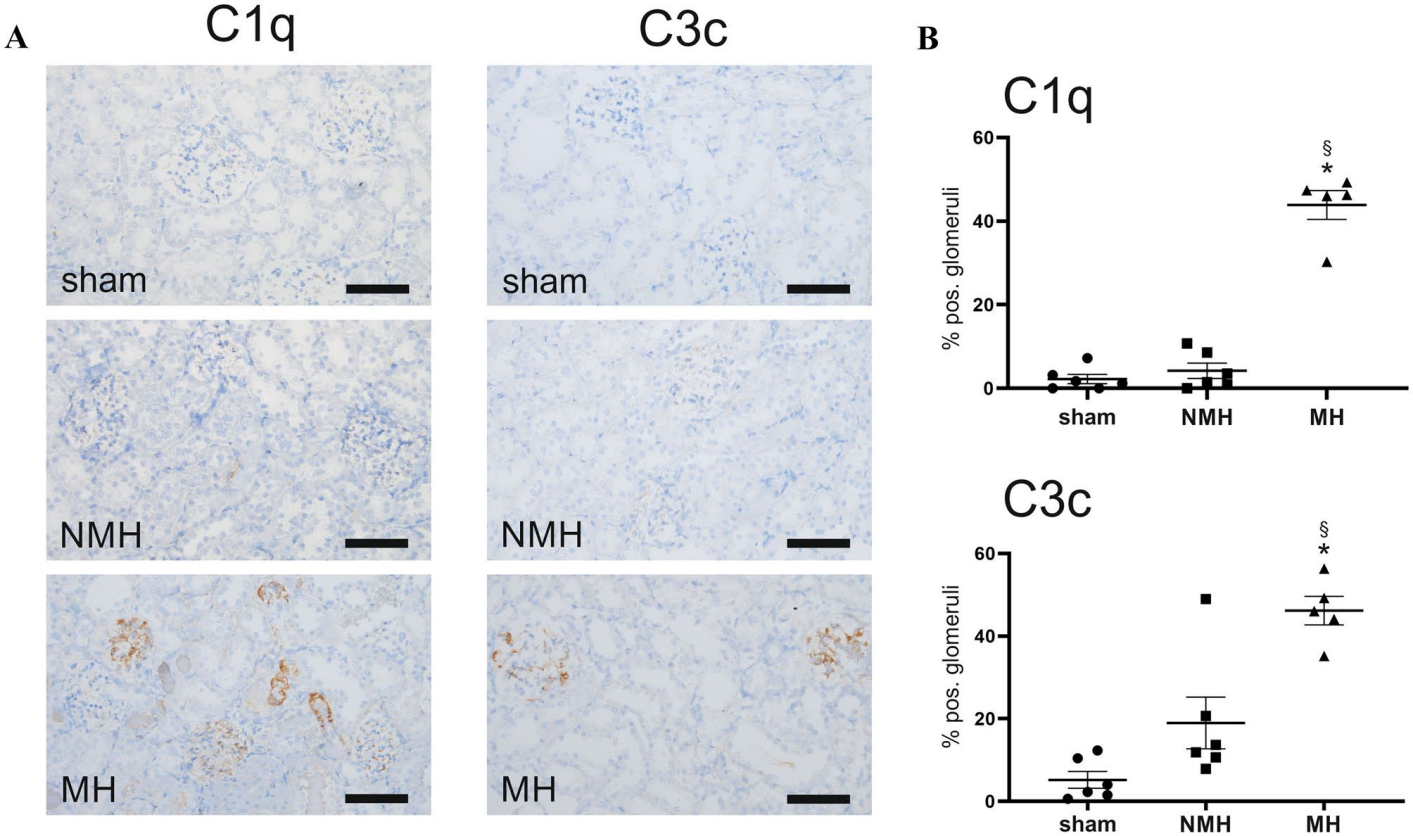
Components of the complement system (C1q and C3c) detected in kidney tissue. **A** Exemplary photomicrographs of renal sections stained for C1q or C3c. Bar represents 100 μm. **B** evaluation of glomeruli stained positive for C1q or C3c. Sham, sham-operated animals (*n* = 6); NMH, non-malignant hypertension (*n* = 6); MH, malignant hypertension (*n* = 5). Data are means ± standard error of the mean. * *p* < 0.05 vs. sham, § *p* < 0.05 vs. NMH

**Fig. 8:**
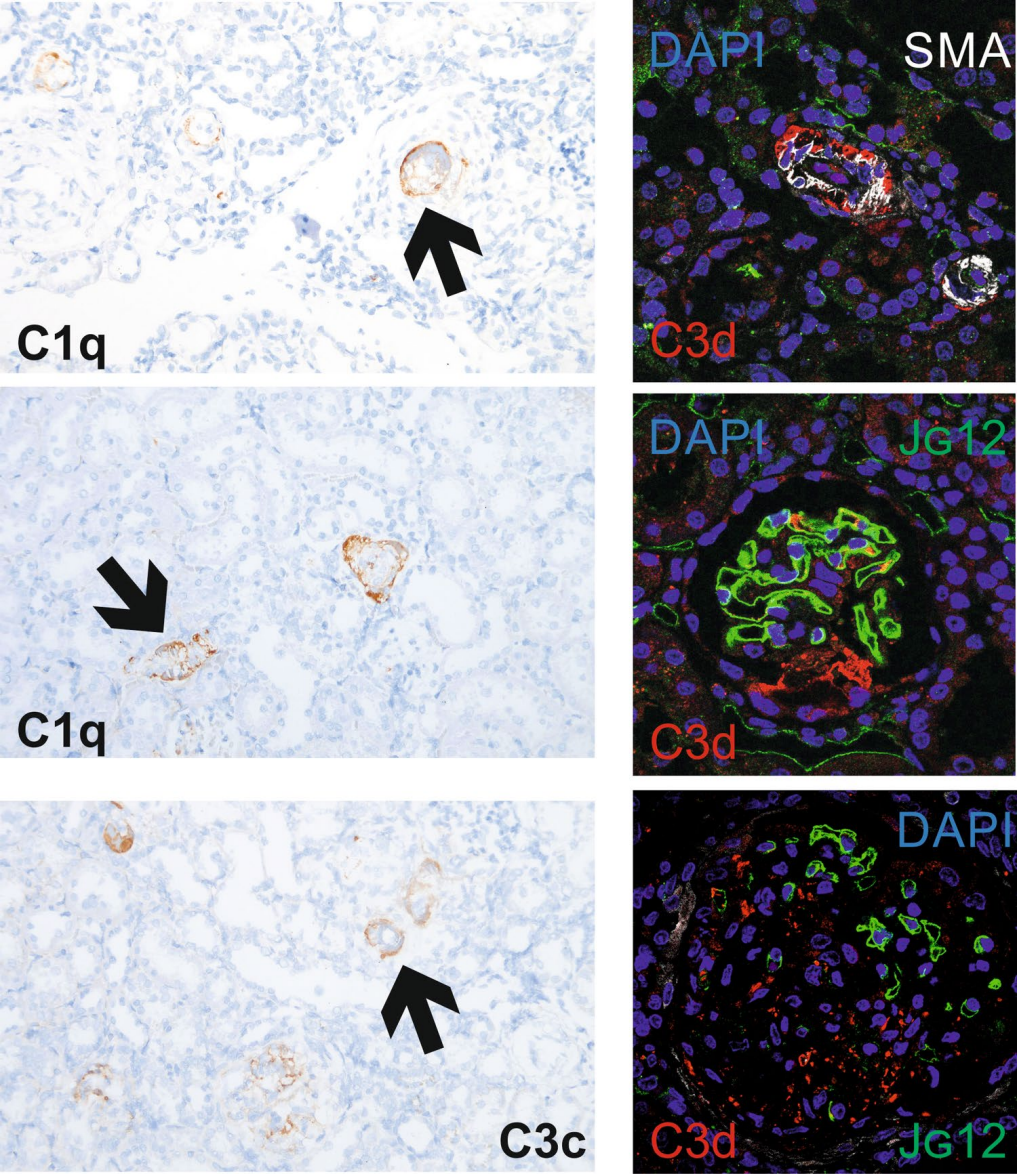
Exemplary photomicrographs of renal sections stained for C1q or C3c. Arrows point to stained vascular structures. Fluorescent stainings to localize complement deposition: Complement C3d (red) was co-stained with α-smooth muscle actin (white, detecting vascular smooth muscle cells) and endothelium of blood vessels (green, aminopeptidase P, see reference 45). Bar represents 50 μm

Using immunohistochemistry, we detected that MH is accompanied by a significantly higher number of C1q and C3c positive glomeruli compared to NMH and sham (Fig. 7). Staining for C1q, C3c, and C3d was also present in damaged preglomerular arterioles in MH (Fig. 8). Immunofluorescence demonstrated that C3d was preferentially localized in areas where endothelial staining was lost, both in glomeruli and arterioles (Fig. 8).

## Discussion

Malignant hypertension is a life-threatening disease. Its pathogenic background is still unclear, especially the factors which determine the development towards malignant or non-malignant hypertension. Apart from that, the transition from non-malignant to malignant hypertension might serve as a model to identify pathways leading to more extensive target organ damage in situations other than malignant hypertension.

In this context, we systematically compared gene expression patterns in our rat model of induced renovascular hypertension leading to the spontaneous development of malignant hypertension in some but not all animals, using RNA-seq. The objective was to detect candidate genes and pathways exclusively regulated under malignant hypertension, compared with non-malignant hypertensive rats. We focused on gene expression in the contralateral (not the clipped) kidney because this kidney displays the characteristic vascular lesions of malignant hypertension. We observed the most prominent upregulation of gene expression in functional pathways related to processes of inflammation and cell infiltration. This result did not come as a surprise, as an inflammatory response in kidneys exposed to high blood pressure has been well recognized for decades in this animal model [13, 19] as well as in other models [20, 21]. Nevertheless, our data point to a number of distinct pathways, which are upregulated in malignant hypertension.

Perhaps the most intriguing finding was the increased local expression of pathway components associated with complement activation. The similarity of renal vascular lesions observed in malignant hypertension to the ones seen in complement-mediated hemolytic-uremic syndrome has often been noted [22], and distinguishing between both diseases can be challenging in the clinical setting. Timmermans et al. [23] have reported that complement abnormalities may underlie at least a subset of patients with malignant hypertension, but this finding has not been corroborated by other authors [24]. In this regard, our model may be of interest because we found both complement activation and vascular lesions suggestive of thrombotic microangiopathy despite the absence of other triggers for complement activation except hypertension. Because the functional role of renal expression of complement factors relative to serum-derived factors (which we did not measure) is unknown, and as the complement cascade is characterized by numerous post-translational interactions of its components, we determined the renal deposition of factors C1 and C3 by immunohistochemistry. Both components were present in glomeruli and preglomerular vessels of malignant hypertensive rats.

Our findings contrast with some animals studies including the report of Cole et al. [25] who did not find an effect of complement C3 deficiency on angiotensin II-induced hypertension and hypertrophy in mice. On the other hand, Ruan et al. [26] described a crucial role of C5a for the vascular inflammation in mouse DOCA-salt hypertension, and Negishi et al. [27] reported that C3 contributes to salt-sensitive hypertension in spontaneously hypertensive rats. Finally, the group of Wenzel et al. [28, 29] described a functional role of the complement receptor C5aR1 for the development of kidney injury in a mouse model of angiotensin II-induced hypertension. Thus, the role of complement factors for hypertensive target organ damage remains controversial. Our results do not permit to distinguish between local complement activation and trapping of complement factors, but the localization of these factors, and their association with serious kidney injury, is compatible with a contribution of complement activity to the most severe forms of vascular and glomerular damage in malignant hypertension.

A number of chemokines known to attract mononuclear cells were induced by renovascular hypertension and even more increased in the malignant form of the disease, notably CCL2 and CCL7. This observation as well as the corresponding increase of M1 macrophage infiltration was to be expected from previous reports from the same model [13]. Others have previously shown that blockade or deficiency of CCL2 ameliorates mononuclear cell infiltration and target organ damage in renovascular or other forms of hypertension [30, 31]. These data are certainly compatible with a role for macrophage infiltration in the development of malignant hypertension, but the parallel increase of M2 macrophage infiltration should not be overlooked. Recent data from single-cell sequencing studies have described a far more complex typology of resident macrophage populations than our relatively crude CD68 (ED1) and CD163 stainings can resolve [32].

We did not observe a marked difference of T-cell infiltration between malignant and non-malignant hypertension, somewhat to our surprise. However, this absence of a difference should not be misunderstood to militate against the role of lymphocytes for the development of high blood pressure in angiotensin II-dependent forms of hypertension, an issue, which has attracted great interest in recent years [33–35]. We focused on the different organ damage between two courses of hypertension (malignant versus non-malignant) with similar blood pressure trajectories, not on the mechanisms leading to high blood pressure per se. Of note, our observations point to a possible role of one pathway involving T-cells that has so far received relatively little attention in the setting of hypertensive target organ damage: co-stimulation; the expression of both CTLA-4 and its binding partner CD80 was increased in malignant hypertension. Vinh et al. reported that inhibition or genetic ablation of co-stimulation prevented the development of experimental hypertension in mice [36]. We are not aware of further studies addressing the role of co-stimulation for target organ injury in established hypertension. One might speculate that the enhanced expression of CTLA-4 and CD80 in malignant hypertension could point to an ongoing specific immune response.

Further, we noted a markedly increased expression of the chemokine CXCL6, which is known to attract neutrophil granulocytes, accompanied by increased expression of E-selectin [37]. Therefore, we attempted to assess granulocyte infiltration by staining for MPO; MPO-positive cells were observed exclusively in kidneys from animals with malignant hypertension. However, some macrophages are also MPO-positive; and we could ultimately not resolve which part of MPO-positive cells were granulocytes versus macrophages. Most previous reports on the role of granulocytes in hypertension focus on circulating neutrophils [38], and neutrophil granulocytes can damage endothelial cells in vitro [39, 40].

Our study has several limitations. First, the study groups MH and NMH might also be regarded as two extremes of a continuous spectrum rather than two distinct clinical courses. Data obtained from PCA as well as the fact that the same intervention by 2K1C induces significantly different courses of pathology supports the classification of MH and NMH animals as two distinct categories. Furthermore, comparisons between two extremes of a spectrum might still point to pathways associated with more severe hypertensive damage. Second, renal alterations in MH animals were only assessed at one time point. This does not allow to discriminate if the observed alterations are cause or effect of malignant hypertension. Our data are fully compatible with the notion of a threshold pressure which might trigger the observed transcriptional changes [5]. Blood pressure was measured by tail cuff during the course and intraarterially at the termination of the experiment, but not by radiotelemetry which might have provided more information. Data obtained by RNA-seq were confirmed by RT-PCR and immunohistochemistry, but no functional tests were performed. These tests will be necessary to clarify in further studies the pathogenic link and mechanistic background of renal damage and malignant hypertension. Further, end-organ damage under malignant hypertension was only examined in the kidney not in other organs also affected by malignant hypertension, e.g., the brain and heart. Finally, we performed bulk RNA-seq with RNA extracted from renal cortical tissue. Single-cell RNA sequencing which was not available to us might have provided more information on the cell infiltrate as well as on the question which pathways are activated in which specific cell type. Conversely, our focus on kidney cortical tissue will miss differential gene expression in kidney medulla. Nevertheless, our data point to potential therapeutic targets for malignant hypertension, especially with regard to the complement system.

## Material and methods

### Induction of renovascular hypertension

All procedures performed on animals were done in compliance with the DIRECTIVE 2010/63/EU of the European Parliament and were approved by the local government authorities (Regierung of Mittelfranken, AZ54-2532.1–51/12). Animal experiments were reported with adherence to the ARRIVE guidelines [41].

Rats were housed in a room maintained at 22 ± 2 °C, exposed to a 12 h dark/light cycle. The animals were allowed unlimited access to chow (#1320, Altromin, Lage, Germany) and tap water. Two-kidney, one-clip renovascular hypertension (2K1C) was induced in male Sprague–Dawley rats (Charles River, Sulzfeld, Germany) weighing 150–170 g by placing a silver clip of 0.2 mm internal diameter around the left renal artery through a flank incision under isoflurane anesthesia as previously described [15]. Control animals underwent sham operation without placement of the clip. Analgesia with subcutaneous buprenorphine injections was provided post-operatively in all animals and as needed later on.

### Experimental groups

Five weeks after clipping of the left renal artery, the experiment was terminated, animals were weighted, and renal tissue was studied for the presence of onion skin lesions and fibrinoid necrosis in all contralateral kidneys exposed to high blood pressure. Thirteen control animals underwent sham operation (sham). Fourteen animals were defined as malignant hypertensive (MH) and 13 as non-malignant hypertensive (NMH). The criteria used for the definition of malignant hypertension in this study (weight loss and characteristic vascular lesions) are described in detail elsewhere [14].

### Blood pressure measurements and determination of serum markers

Procedures of blood pressure measurements were described previously [14, 42]. In short, systolic blood pressure values were obtained by tail cuff measurements in trained rats 1, 1.5, 2, 3, 4, and 5 weeks after 2K1C using a peripheral blood pressure monitoring system (TSE Technical Scientific Equipment GmbH, Bad Homburg, Germany). At the end of the experiment, femoral artery catheters were implanted under isoflurane anesthesia for invasive blood pressure measurements. Measurements were performed in conscious animals via transducers connected to a polygraph (Hellige, Freiburg, Germany). Blood samples with a total volume of 2 ml were collected immediately before euthanasia. Serum creatinine, urea, and aldosterone were analyzed using the automatic analyzer Integra 800 (Roche Diagnostics, Mannheim, Germany).

### Tissue preparation and histological analysis

After organ weighing, kidneys were decapsulated. Both poles of each kidney were immediately snap frozen on liquid nitrogen for RNA extraction. One slice of the kidney was snap frozen for protein isolation, while another slice of the remaining kidney was put in Methyl Carnoy’s fixative (60% methanol, 30% chloroform, and 10% glacial acetic acid) for fixation. Paraffin-embedded tissue was sectioned and stained with periodic acid Schiff’s (PAS) reagent for detection of onion skin lesions and fibrinoid necrosis.

### RNA isolation

RNA was isolated from tissue samples after homogenization with a disperser (T10 basic Ultra-Turrax; IKA, Staufen, Germany) using the RNeasy Fibrous Tissue Midi Kit (Qiagen, Hilden, Germany). In this procedure, Proteinase K was used as well as DNase to remove genomic DNA. The entire process was carried out according to the manufacturer’s instructions.

### Whole transcriptome analysis (RNA-seq)

As reported previously, quality of isolated RNA samples was determined using the Agilent 2100 Bioanalyzer equipped with an Agilent RNA 6000 Nano kit and related software (Agilent, Santa Clara, CA) [43]. RNA integrity number (RIN) values of ≥ 5 were deemed suitable for analysis [44]. The RIN values of the samples were in a range from 6.5 to 10.5.

RNA-seq was performed in MH (*n* = 6), NMH (*n* = 5), and sham animals (*n* = 5). Sequencing libraries were generated from 0.5 ug high-quality RNA using the TruSeq Stranded mRNA Kit (Illumina, San Diego, USA) according to the manufacturer’s instructions. Libraries were sequenced on a HiSeq 2500 platform (Illumina, San Diego, USA) as 101 bp single-end reads to a depth of at least 25 million reads. Reads were converted to FASTQ format while masking adapter sequences (bcl2fastq v2.17.1.4, Illumina, San Diego, USA). Sequences mapping to rRNA, tRNA, mtRNA, and transposons were removed by alignment against a custom reference list (bwa v0.7.14, samtools v1.8). Remaining reads were mapped to the *Rattus norvegicus* reference genome Rnor 6.0, Ensembl gene annotation 93, using a splice-aware aligner (STAR v2.5.4a) and quantified as reads per gene while excluding exons shared between more than one gene (subread v1.5.3). Based on this quantification, differentially expressed genes were determined using the negative binomial model as implemented in DESeq2 (DESeq2 v1.20.0, R v3.5.0). Analysis was performed in the following groups: MH vs. NMH, NMH vs. sham, MH vs. [NMH + s ham], and [MH + NMH] vs. sham. Results from significance tests were corrected for multiple testing (Benjamini-Hochberg).

Raw data for RNA-seq are publicly available in the Gene expression Omnibus (GEO, NCBI) repository under the accession number (pending).

### Network and pathway analysis

In silico network and pathway analysis was performed using Ingenuity Pathway Analysis software, Version 52,912,811 (Qiagen, Hilden, Germany). Differentially expressed genes with log2 fold change (LFC) > ± 2 and a corrected *p* value < 0.01 were analyzed. By the comparison with the current Ingenuity knowledge base significantly altered networks, upstream regulators and central candidate genes were extracted.

### Immunohistochemistry

Tissue was processed as described previously [14]. In summary, three micron sections of renal tissue were cut with a Leitz SM 2000 R microtome (Leica Instruments, Nussloch, Germany). Endogenous peroxidase activity was blocked with 3% H2O2 in methanol for 20 min at room temperature. Antigen retrieval was performed using Target Retrieval Solution (TRS) (DAKO, Hamburg, Germany). For the primary antibody, an overnight incubation at 4 °C was chosen. Secondary antibodies were incubated for 2 h at room temperature. Antibodies against CD68 (ED1; AbD Serotec, Kidlington; UK 1:250), myeloperoxidase (MPO) (Abcam, Cambridge, UK; 1:50), CD3 (Abcam, Cambridge, UK; 1:50), CD4 (Cell signaling, Danvers, Massachusetts, USA; 1:50), CD8a (Abcam, Cambridge, UK; 1:50), CD163 (Abcam, Cambridge, UK; 1:50), C1q (Dako, Glostrup, Denmark; 1:75,000), and C3c (Dako, Glostrup, Denmark; 1:75,000) were used. Infiltration by inflammatory cells was assessed after staining with CD68 (M1 macrophages), MPO (neutrophil granulocytes, some macrophages), CD3 (T-lymphocytes), FoxP3 (regulatory T-cells), CD4 (T-helper cells), CD8a (cytotoxic T-cells), and CD163 (M2 macrophages) by counting the number of positive cells in 20 medium-power views (magnification × 200 on a Leitz microscope). Furthermore, in each kidney, 100–200 glomeruli were counted, and the number of C1q and C3c positive glomeruli was expressed as a percentage of the total number of glomeruli counted. All antibodies were used on paraffin-embedded tissue sections. For immunofluorescence stainings antibodies to α-smooth muscle actin (DAKO, Hamburg, Germany), aminopeptidase P (Invitrogen, Karlruhe, Germany) [45] and C3d (Abcam, Cambridge, UK) were applied, followed by secondary fluorescent antibodies: goat anti-mouse IgG1 conjugated with Alexa Fluor 488 (Dianova, Hamburg, Germany) and goat anti-mouse IgG2a conjugated with Alexa Fluor 633 (Life technologies GmbH, Darmstadt, Germany); sections were covered with autofluorescence quenching kit TrueView (Vector Laboratories, Burlingame, CA, USA) and analyzed using a confocal laser scanning microscope (LSM710) and ZEN software (both Zeiss, Oberkochen, Germany) All histological evaluations were done by a single investigator blinded to the group assignment.

### Real-time polymerase chain reaction (PCR) analyses

Renal and myocardial tissue was homogenized in RLT buffer reagent (Qiagen, Hilden, Germany) with an ultraturrax for 30 s, total RNA was extracted from homogenates by RNeasy Minicolumns (Qiagen) according to the manufacturer’s protocol, and real-time RT-PCR was performed. First-strand cDNA was synthesized with TaqMan reverse transcription reagents (Applied Biosystems, Darmstadt, Germany) using random hexamers as primers. Reactions without Multiscribe reverse transcriptase were used as negative controls for genomic DNA contamination. PCR was performed with an ABI PRISM 7000 Sequence Detector System and TaqMan or SYBR Green Universal PCR Master Mix (Applied Biosystems), as described previously [42]. All samples were run in triplicates. Specific mRNA levels in hypertensive animals relative to sham-operated controls were calculated and normalized to a housekeeping gene (18S) with the Δ-Δ-C_T_ method as specified by the manufacturer (http://www3.appliedbiosystems.com/cms/groups/mcb_support/documents/generaldocuments/cms_040980.pdf). Primer pairs used for experiments are shown in supplementary table 4.

## Statistics

Data are expressed as means ± standard error of the mean (SEM). Normality was tested using the Shapiro–Wilk test. Subsequently, to assess differences between sham, NMH, and MH animals, one-way analysis of variance (one-way ANOVA), followed by Bonferroni post hoc test or, where appropriate, Kruskal–Wallis, followed by Dunn’s test, was performed using IBM SPSS 21 (SPSS Inc. Chicago, IL, USA). Results were considered significant at *p* < 0.05. Pearson correlations were used to assess associations between selected molecular markers of inflammation and inflammatory cells. Strength of Pearson’s correlations was graded according to the following pattern: *r* = 0.0–0.19 “very weak,” *r* = 0.2–0.39 “weak,” *r* = 0.4–0.59 “moderate,” *r* = 0.6–0.79 “strong,” and *r* = 0.8–1.0 “very strong.” Statistical significance was set at *p*-values < 0.05 [46].

## Data availability statement

Raw data for RNA-seq supporting this study are publicly available in the Gene expression Omnibus (GEO, NCBI) repository at https://www.ncbi.nlm.nih.gov/geo/query/acc.cgi?acc=GSE154019.

## Supplementary Information

Below is the link to the electronic supplementary material.

Supplementary file1 (DOCX 15465 KB)

Supplementary file2 (DOCX 19 KB)

## Declarations

### Statement on welfare of animals

All procedures performed on animals were done in compliance with the DIRECTIVE 304 2010/63/EU of the European Parliament.
