# Supplementary material for: RNA sequencing reveals induction of specific renal inflammatory pathways in a rat model of malignant hypertension
Source: J Mol Med (Berl). 2021 Sep 15;99(12):1727–40. doi: 10.1007/s00109-021-02133-8 (PMC8599225; doi:10.1007/s00109-021-02133-8)
Supplement: Supplementary file 1 — Supplementary file1 (DOCX 15465 KB) [file 109_2021_2133_MOESM1_ESM.docx]

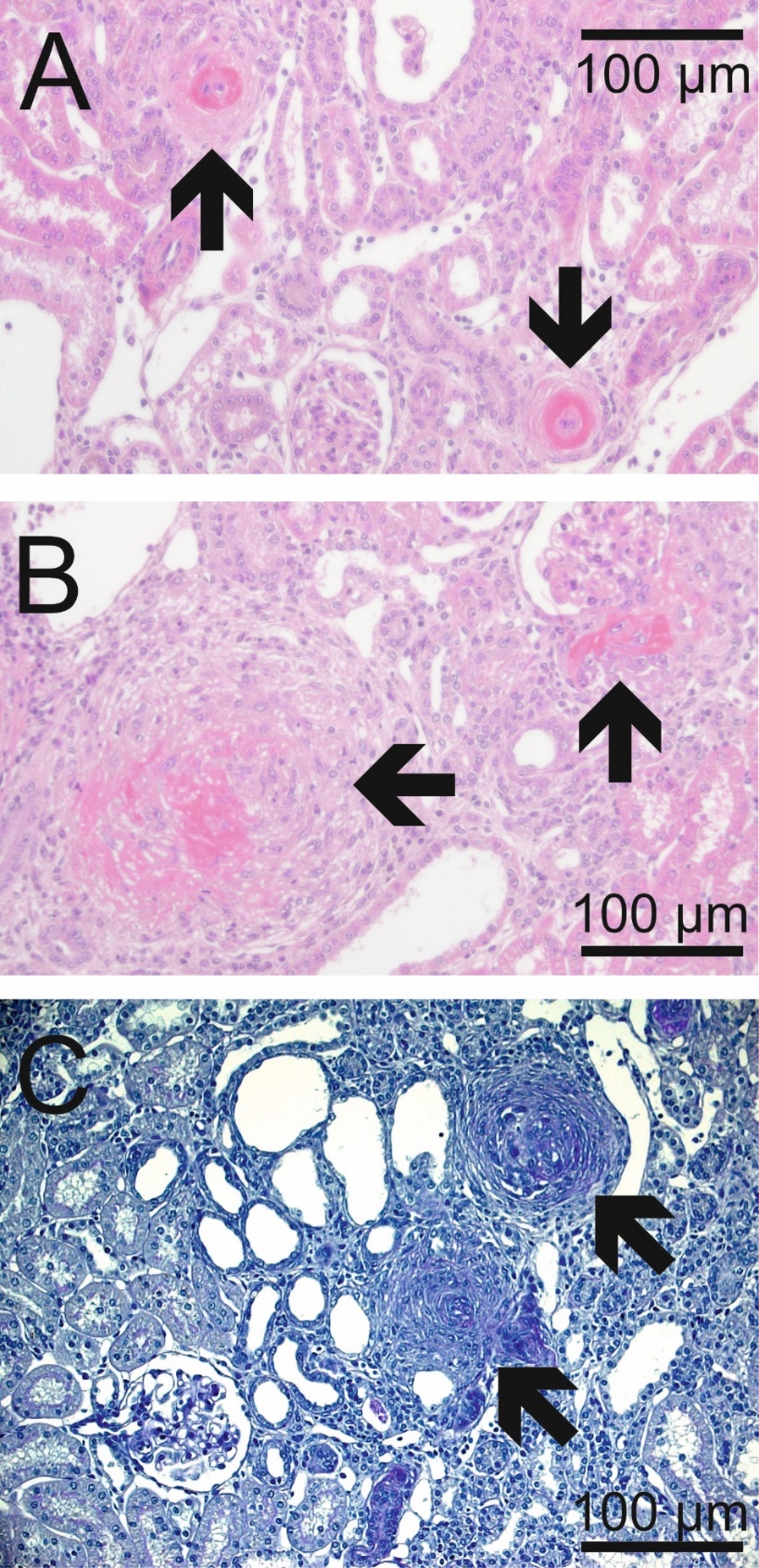


**Supplementary Fig. 1** HE or PAS-stained renal sections of animals with malignant hypertension A, B arrows point to fibrinoid necrotic lesions. C, arrows mark onion skin lesions. Bar represents 100 μm.


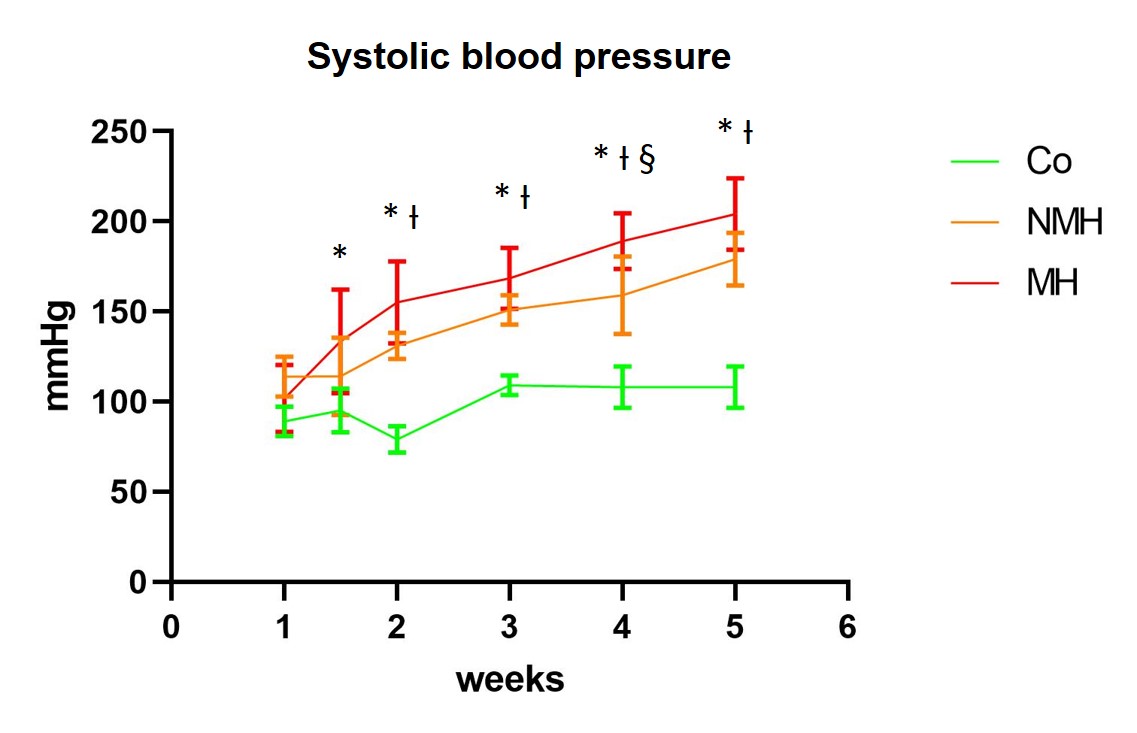


**Supplementary Fig. 2** Systolic blood pressure values obtained by sequential tail-cuff measurements. Sham, sham operated animals, NMH, non-malignant hypertension, MH, malignant hypertension. Data are means ± standard error of the mean. * p < 0.05 MH vs. sham, Ɨ p < 0.05 NMH vs. sham, § p < 0.05 MH vs NMH.


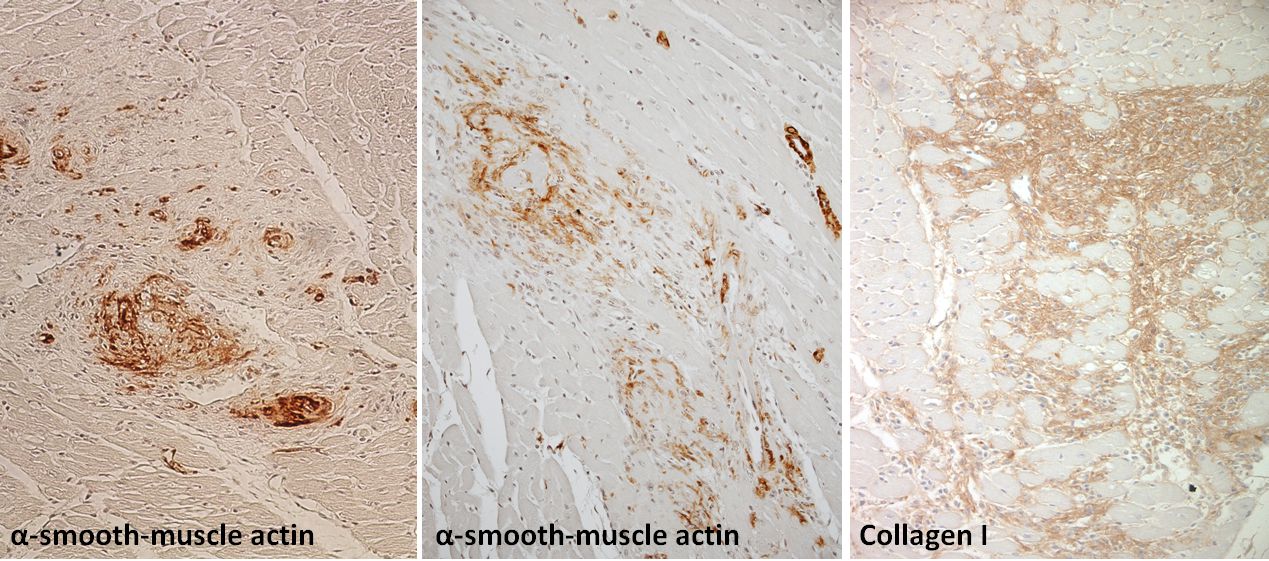


**Supplementary Fig. 3** Exemplary photomicrographs of the myocardium of malignant hypertensive animals stained for α-smooth muscle actin or collagen type !.


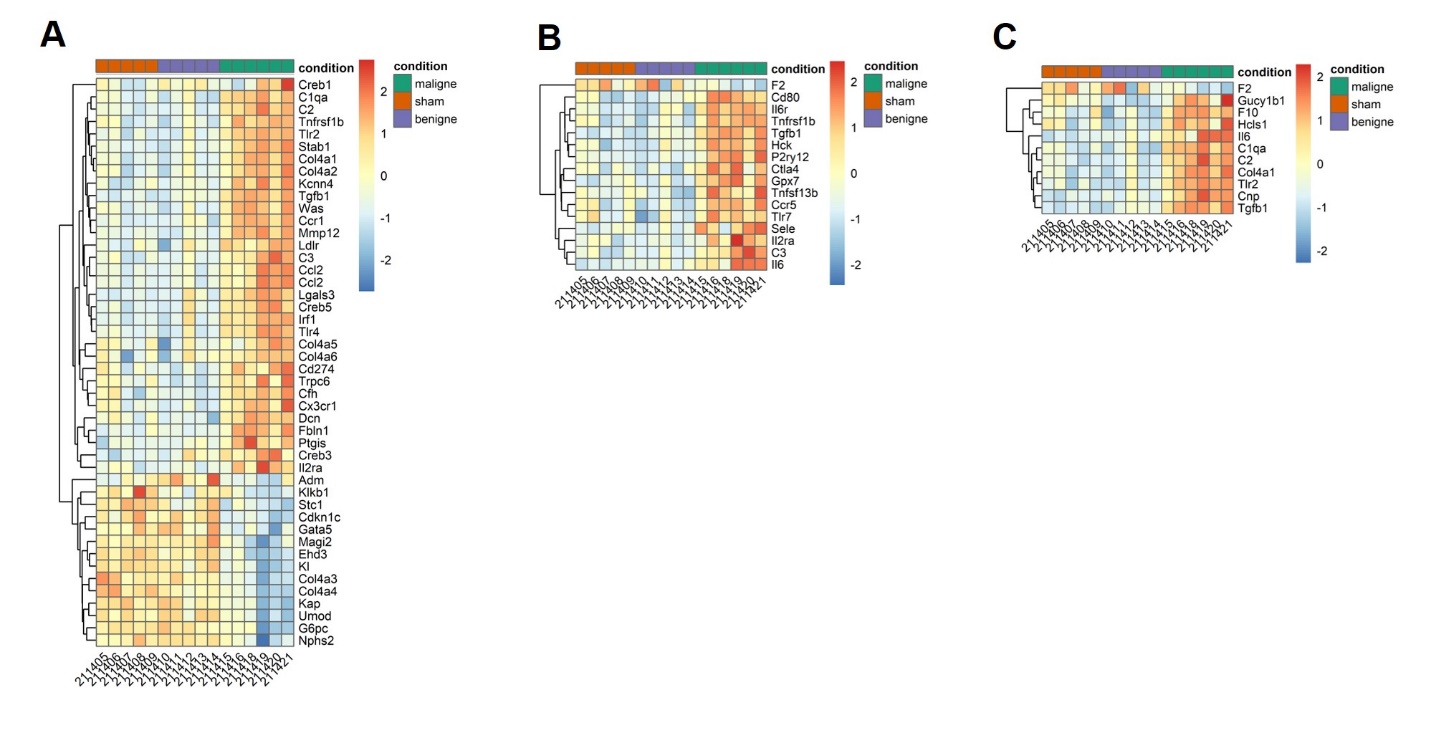


**Supplementary Fig. 4** Unclustered heat map analyses of normalized (rlog, DESeq2) expression values, centered and scaled by row. A, heat map with genes related to “glomerular disease”; B, heat map with genes related to “vasculitis”; C, heat map with genes related to “thrombosis”.


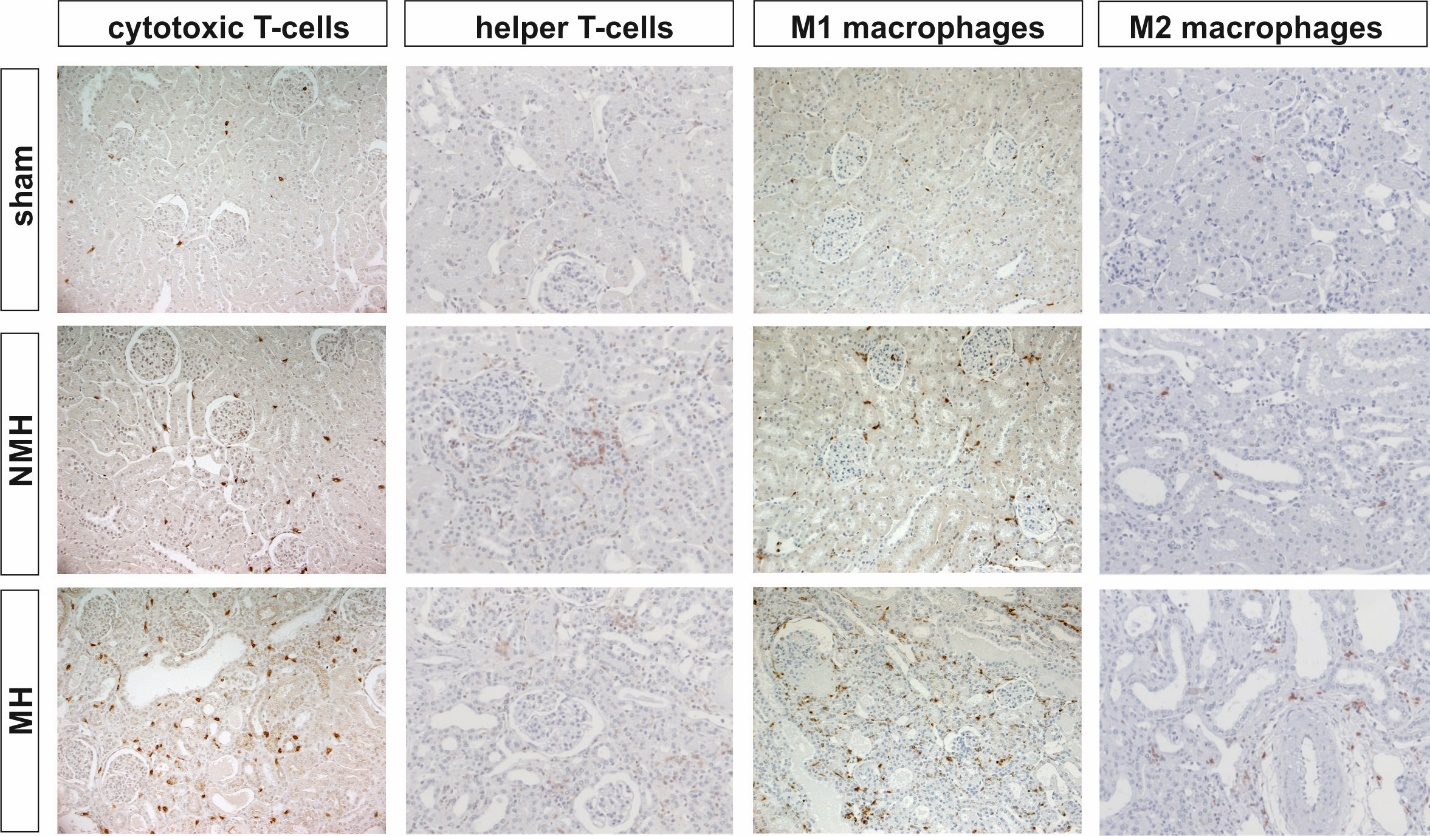


**Supplementary Fig. 5** Infiltration of cytotoxic T-cells, helper T-cells, M1 and M2 macrophages Exemplary photomicrographs of renal sections stained for CD8a, CD4, CD68 and CD163. Sham, sham operated animals, NMH, non-malignant hypertension, MH, malignant hypertension.


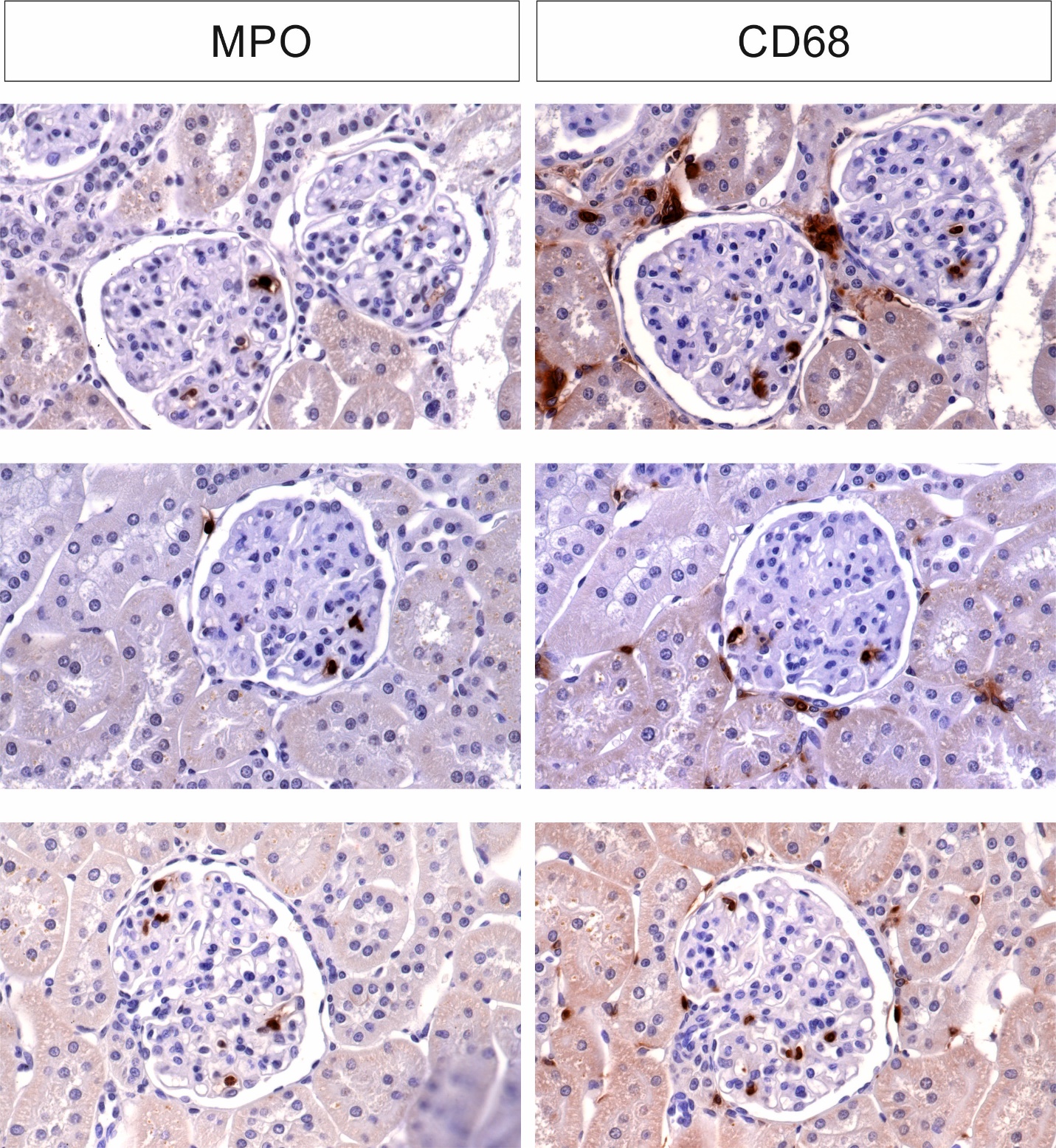


**Supplementary Fig. 6** Serial sections of renal cortical tissue stained for myeloperoxidase (MPO) or CD68.


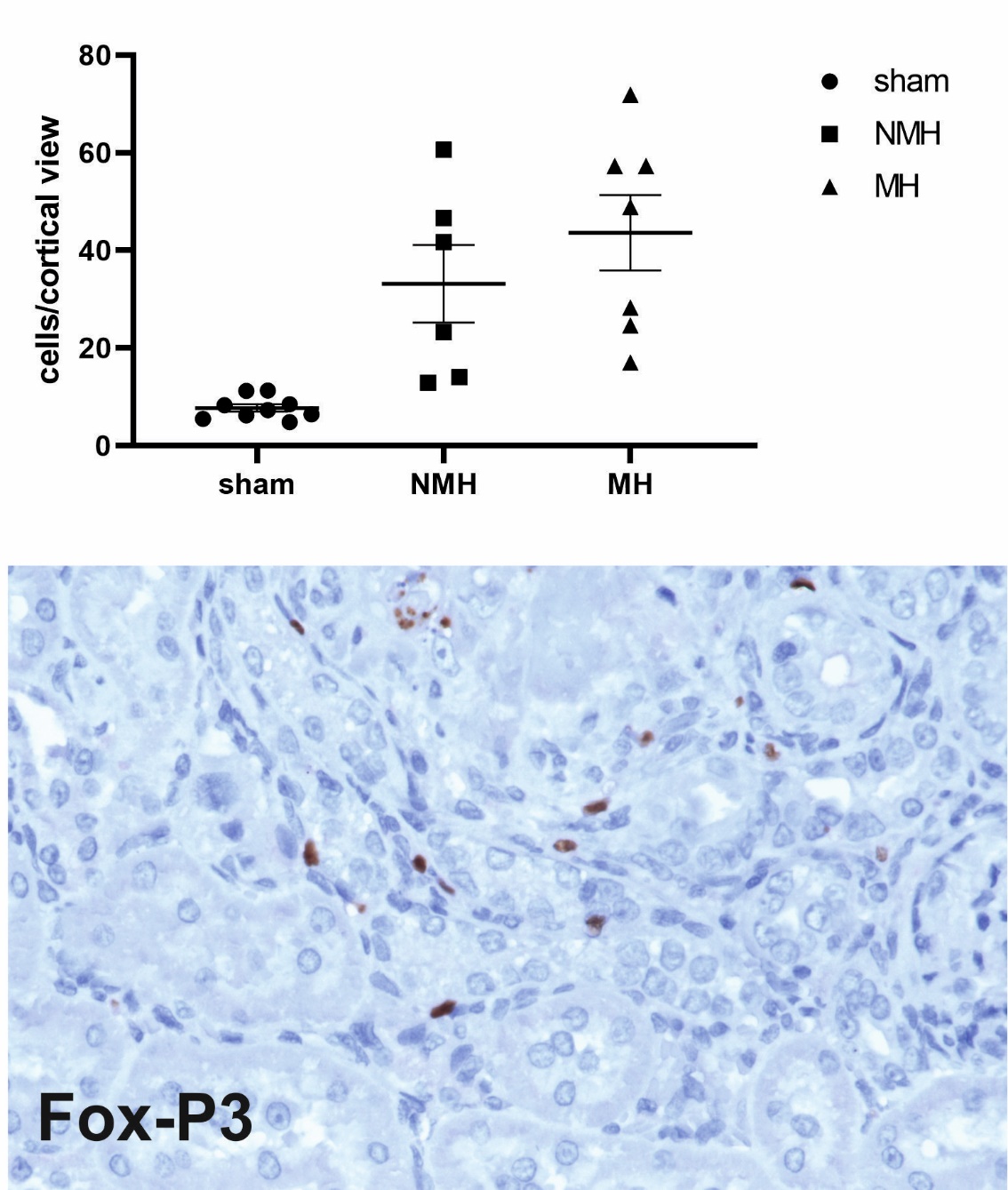


**Supplementary Fig. 7** Infiltration of regulatory T-cells. Evaluation of cell counts in renal sections and exemplary photomicrograph of FoxP3-stained cortical tissue. Sham, sham operated animals, NMH, non-malignant hypertension, MH, malignant hypertension.
