# Supplementary material for: RNA sequencing reveals induction of specific renal inflammatory pathways in a rat model of malignant hypertension
Source: J Mol Med (Berl). 2021 Sep 15;99(12):1727–40. doi: 10.1007/s00109-021-02133-8 (PMC8599225; doi:10.1007/s00109-021-02133-8)
Supplement: Supplementary file 2 — Supplementary file2 (DOCX 19 KB) [file 109_2021_2133_MOESM2_ESM.docx]

**Supplementary table 1: Data from animals included in RNA-seq analysis**

|  | **Sham (n=5)** | **NMH (n=5)** | **MH (n=6)** |
| --- | --- | --- | --- |
| Weight [g] | 405.3±13.7 | 357.2±12.7 | 244.8±11.9 *^§^ |
| Right Kidney Weight [g] | 1.29±0.04 | 1.35±0.12 | 1.61±0.15 |
| Relative RKW [mg/g] | 3.12±0.08 | 3.81±0.41 | 6.25±0.59 *^§^ |
| Serum Urea [mg/dl] | 36.62±1.61 | 39.72±4.77 | 79.23±8.10 *^§^ |
| Serum Crea [mg/dl] | 0.19±0.02 | 0.21±0.01 | 0.33±0.04 *^§^ |
| Serum Aldosterone [pg/ml] | 245.3±58.6 | 1091.9±416.0 | 4453.4±1072.2 *^§^ |
| MAP [mmHg] | 119.5±1.7 | 192.0±6.4 * | 220.0±6.5 *^§^ |
| Left Ventricular Weight [g] | 0.80±0.03 | 1.06±0.04 * | 0.92±0.04 |
| Relative LVW [mg/g] | 1.98±0.03 | 2.98±0.18 * | 3.59±0.20 * |

* p-value < 0.05 versus sham, § p-value < 0.05 versus NMH

**Supplementary table 2: Urine analysis obtained from animals of an additional cohort kept in metabolic cages 24 hours before sacrifice**

|  | **Sham (n=9)** | **NMH (n=5)** | **MH (n=7)** |
| --- | --- | --- | --- |
| Albuminuria [mg/24h] | 0.48±0.28 | 35.4±10.6 | 109.9±19.4 *^§^ |
| Proteinuria [mg/24h] | 14.0±12.7 | 45.1±14.6 | 113.0±23.5 * |

* p-value < 0.05 versus sham, § p-value < 0.05 versus NMH

**Supplementary table 3: Correlation of markers of renal damage, inflammation and fibrosis with Complement C3 expression in 2K1C**

| **Complement C3** (mRNA expression) |  | |
| --- | --- | --- |
|  | **r** | **p-value** |
|  |  |  |
| **Serum creatinine** (mg/dl) | 0.75 | **0.001** |
| **Serum urea** (mg/dl)  **Mean arterial blood pressure** (mm Hg)  **KIM1** (mRNA expression)  **Cb4** (mRNA expression)  **C6** (mRNA expression)  **C5aR1** (mRNA expression)  **MPO-positive cells** (cells/view) | 0.83  0.16  0.79  0.88  0.62  0.79  0.73 | **< 0.001**  0.58  **0.001**  **< 0,001**  **0.013**  **<0.001**  **0.005** |
| **C1q stain** (% pos. glomeruli) | 0.94 | **0.005** |
| **C3c stain** (% pos. glomeruli) | 0.97 | **< 0.001** |

r = Spearman-Rho correlation coefficient r

Statistical significance was defined as p-value < 0.05

**Supplementary Table 4: List of primer pairs and probes used in the study**

|  | Forward | Reverse |
| --- | --- | --- |
| Complement C3 | 5‘-CAGCCTGAATGAACGACTAGACA-3‘ | 5‘-ACGTAGTCCACTCCAGGCTCA-3‘ |
| Complement C3aR1 | 5‘-ATCCCACCTCAGTGCTCTTGA-3‘ | 5‘-GTGTCAGCAGTGAAAGACTCCATT-3‘ |
| Complement C4b | 5‘-CCGAGTGGAGTATGGCTTCC-3‘ | 5‘-GATCTGCTGTCTTCTCGGAGAAC-3‘ |
| Complement C5aR1 | 5‘-CAGGAGAAGCCAGGACATG-3‘ | 5‘-TGATTTCACTGCTGTCGTTACTTATG-3‘ |
| Complement C6 | 5‘-GTCCAGCTGTTCTAAGTCCTGCA-3‘ | 5‘-TGTCTCTGTCTGCTCTGGGTTC-3‘ |
| CCL2 | 5‘-CCTCCACCACTATGCAGGTCTC-3‘  probe: 5‘-TCACGCTTCTGGGCCTGTTGTTCA-3‘ | 5‘-GCACGTGGATGCTACAGGC-3‘ |
| CCL3 | 5‘-TCCTGCCACCTGCAAATCTC-3‘ | 5‘-GCTACTTGGCAGCAAACAGCT-3‘ |
| CCL5 | 5‘-GTCGTCTTTGTCACTCGAAGGA-3‘ | 5‘-GATGTATTCTTGAACCCACTTCTTCTC-3‘ |
|  | probe: 5‘-CCGCCAAGTGTGTGCCAACCC-3‘ | |
| CCL7 | 5‘-GCCGCGCTTCTGTGTGT-3‘  probe: 5‘-CTGCTCACAGCTGCTGCTTTCACCG-3‘ | 5‘-TGGATGAATTGGTCCCATCTG-3‘ |
| CXCL3 | 5‘-TTTTGAGAACATCCAGAGCTTGAC-3‘ | 5‘-CTTGAGAGTGGCTATGACTTCTGTCT-3 |
| CXCL6 | 5‘-CACACTGCCACAGCATCGA -3‘ | 5‘-CAGCGTAGCTCCGTTGCAA-3‘ |
| CXCL8 | 5‘-CCTTCCTCATTTTTGCAGCTACTC -3‘ | 5‘-CATGTACGATCTGGGCCTGG-3‘ |
| CCR2 | 5‘-CTGTGTGGTTGACATGCACTTAGA -3‘  probe: 5‘-AGACTCTTGGAATGACACACTGCTGCGTTA-3‘ | 5‘-ACTCGGTCTGCTGTCTCCCTATAG -3‘ |
| IL-6 | 5‘-GCCCTTCAGGAACAGCTATGA-3‘  probe: 5‘-TCTCCGCAAGAGACTTCCAGCCAGTT-3‘ | 5‘-TGTCAACAACATCAGTCCCAAGA-3‘ |
| IL-10 | 5‘-GCTGTCATCGATTTCTCCCC-3‘ | 5‘-CCTGCTCCACTGCCTTGC-3‘ |
| IL-17a | 5‘-CACAAGCTCATCCCGTACCA-3‘ | 5‘-CAGGCACATGGATGGAATTCT-3‘ |
| LIF | 5‘-ACGGCAACCTCATGAACCA-3‘ | 5‘-TGTGTAATAGGAAATAAAGAGGGCAT-3‘ |
| TNF-α | 5‘-ATGGGCTCCCTCTCATCAGT-3‘ | 5‘-GCTTGGTGGTTTGCTACGAC-3 |
| ICAM-1 | 5‘-GGGCCCCCTACCTTAGGAA-3‘ | 5‘-GGGACAGTGTCCCAGCTTTC-3 |
| VCAM-1 | 5‘-TGTGGAAGTGTGCCCGAAAT-3‘ | 5‘-TGCCTTGCGGATGGTGTAC-3 |
